# Stimuli-Directed Helical Chirality Inversion and Bio-Applications

**DOI:** 10.3390/polym8080310

**Published:** 2016-08-18

**Authors:** Ziyu Lv, Zhonghui Chen, Kenan Shao, Guangyan Qing, Taolei Sun

**Affiliations:** 1 State Key Laboratory of Advanced Technology for Materials Synthesis and Processing, Wuhan University of Technology, 122 Luoshi Road, Wuhan 430070, China; lvziyu@whut.edu.cn (Z.L.); chenzhonghui@whut.edu.cn (Z.C.); meou2016720@whut.edu.cn (K.S.); 2 School of Chemistry, Chemical Engineering and Life Science, Wuhan University of Technology, 122 Luoshi Road, Wuhan 430070, China

**Keywords:** helical chirality, stimuli-responsive, supramolecular assembly, helical polymer

## Abstract

Helical structure is a sophisticated ubiquitous motif found in nature, in artificial polymers, and in supramolecular assemblies from microscopic to macroscopic points of view. Significant progress has been made in the synthesis and structural elucidation of helical polymers, nevertheless, a new direction for helical polymeric materials, is how to design smart systems with controllable helical chirality, and further use them to develop chiral functional materials and promote their applications in biology, biochemistry, medicine, and nanotechnology fields. This review summarizes the recent progress in the development of high-performance systems with tunable helical chirality on receiving external stimuli and discusses advances in their applications as drug delivery vesicles, sensors, molecular switches, and liquid crystals. Challenges and opportunities in this emerging area are also presented in the conclusion.

## 1. Introduction

Chirality represents an important biochemical signature of life, which exists widely in nature and plays fundamental but critical roles in the bioactivities of biomolecules and a wide diversity of biochemical reactions [1]. At molecular level, the basic building blocks of biomacromolecules and organisms are homochiral small molecules, such as l-amino acids (except glycine), d-saccharides, and l-phospholipids. The chiral signals of these small molecules are usually very weak, hence, rendering them difficult to be directly associated with physiological functions of biomacromolecules and organisms. Through a bottom-up approach, this molecular chirality can be translated and amplified into supramolecular helical handedness (e.g., secondary alpha-helix structures of peptides and proteins), which is of great importance since it is closely related to molecular recognition, catalytic activity and gene replication in living systems [2,3,4]. The helical superstructures are the central structural motifs in living systems, and also ubiquitous in many artificial materials. As a typical example, a DNA molecule, which carries most of the information in the construction, functioning, and reproduction of all living systems, exhibits an exquisite double helix structure and plays essential roles in gene expression [5]. Interestingly, the helical sense of these macromolecules is not invariable, actually, they exhibit conformational flexibility under certain biological environment in vivo since the driven forces for constructing these helical structures are based on weak interactions (e.g., hydrogen bonding, electrostatic, van der Waals forces) [4,6]. Moreover, living systems utilize a series of complex and elusive helical inversion events of biomacromolecules (e.g., DNA or proteins) to perform various physiological processes, thus achieving numerous specific bio-functions [6,7,8].

For instance, DNA generally exhibits a right-handed helix (e.g., B-DNA), and left-handed Z-DNA was accidentally discovered in the late 1970s. Interesting, helical handedness inversion between low-energy B-DNA and high-energy Z-DNA occurs as one of the most sophisticated conformational changes during physiological processes, which shows great significance in the regulation of gene expression and DNA processing [8,9,10]. Besides, for peptides and proteins, helicity inversion can be also achieved through their flexible peptide bonds. For example, polyproline can adopt helical senses between right-handed and left-handed by means of *cis*-*trans* inter-conversion [11]. In addition, the bacterial flagellar filament, consisting of 11 protofilaments of flagellin, exhibits a bi-stable helical structure. The chiral inversion of this helical architecture between the left- and right-handed supercoiled form happens during a bi-stable mechanical switch of bacteria, in which the corresponding “swimming” pattern alternates between running and tumbling [12].

Inspired by these highly sophisticated biological helices and corresponding helical chirality inversion events, a big challenge and a new direction for chemical and material science, is now how to design artificial systems with controllable helical sense and further use them to mimic natural systems, and develop chiral functional devices as well as promote their applications in biochemistry and nanotechnology fields. In this aspect, stimuli-responsive molecular or polymeric systems provide good solutions for this challenge, in which helical senses could be inversed reversibly under the control of external stimuli, such as solvent, light irradiation, ion, temperature, pH and others [13,14,15]. Chemists and materials scientists have innovatively designed and developed high-performance systems and materials with tunable helical handedness on receiving external stimuli. On the one hand, compared with single molecular conformational modulation, these systems can offer dramatic conformational changes, which provide good candidates to transform external stimuli into the macroscopic properties of materials [14]. On the other hand, these systems can experience accurate right- and left-handed helical inversion in response to external stimuli, which serve as excellent candidates to explore the role of helical chirality in complex biological and chemical processes, and mimic natural systems [4]. Taking advantages of dramatic and mirrored switching in the stimuli-responsive systems, various chiral devices have been developed and find plenty of useful applications in drug delivery [16], asymmetric catalysis [17], molecular motors [18], responsive liquid crystal (LC) materials [15], and other related areas [19,20].

**Figure 1:**
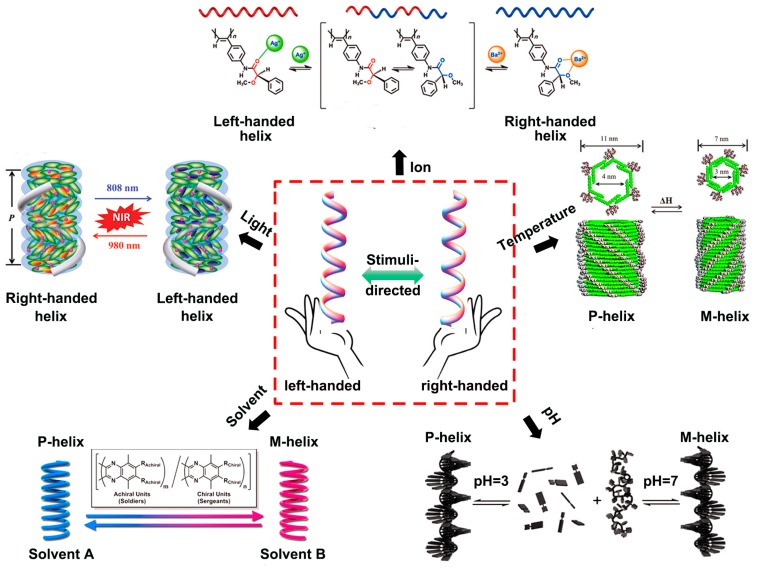
Schematic illustration of helical chirality inversion through different external stimuli, such as solvent, light irradiation, ion, temperature and pH. Typical examples for these modulate methods are also displayed. Reproduced from [21,22,23,24,25] with copyright permission.

In this review, first, we discuss the recent progress over the past decade in the area of helical chirality switching in stimuli-responsive polymer systems. The introduction of stimuli-responsive groups into the side or main chains of helical polymer systems helps to realize reversible switching of helical senses by external stimuli, such as solvent, light irradiation, ion, temperature, and pH. We then introduce the applications of helicity controllable systems in drug delivery, imaging, asymmetric catalysis, molecular machines (e.g., chiral motor, molecular switch), LC materials. The outline of this mini-review is illustrated in Figure 1. Challenges and opportunities in this emerging area are also presented in the conclusion.

## 2. Stimuli-Directed Helical Chirality Inversion

The helical chirality of molecules and supramolecules can be modulated by external stimuli. Here, classified by different stimuli, such as solvent, photo, ion, temperature, and pH, we summarized the helicity switching in this area.

### 2.1. Solvent-Directed Helical Chirality Inversion

Solvent serves as one of the most common sources of stimuli for helical chirality inversion, helical senses can be easily mediated by means of using a variety of organic and inorganic solvents with distinct physical and chemical properties. In particular, some organic solvents are intrinsically chiral, and the chirality of these solvents (e.g., limonene) can significantly influence the solute conformation, consequently, providing a simple and effective method to complete helical chirality switching events. Chemists and material scientists have succeeded in transforming chirality by solvent stimuli, especially the chirality switching for supramolecular and polymeric systems.

Through self-assembly behavior, small molecules can transform and amplify their molecular chirality into delicate helical senses. Interestingly, the helical sense of these molecules can be modulated by solvent stimuli. Martinez et al. prepared an atrane derivative (i.e., vanatrane) with a C_3_-symmetrical backbone which could change reversibly between right- or left-handed chirality in response to deuterated solvent stimuli [26]. In C_6_D_6_ and CDCl_3_, the vanatrane structure displayed an anticlockwise or a clockwise motion, respectively. Based on this solvent stimuli-directed rotational motions of the vanatrane unite, unidirectionally rotational motion of the molecular propeller can be obtained. Kwak et al. achieved a chirality transfer from chiral solvents to polymer main chains by a conjugated polymer [27]. They synthesized a conjugated polymer (poly(diphenylacetylene)s (PDPAs)) with different chain lengths and substitution position of the side alkyl groups, which exhibited intramolecular stack structures. Through a pair of enantiomeric solvent (i.e., *R*/*S*-limonene) stimuli, the induction and inversion of helical sense could be completed because of the discrepant intermolecular interactions between the polymer side chains and the enantiomeric solvents.

Interestingly, supramolecular chirality switching of helical nanotubes has been also achieved by solvent stimuli. Fenniri et al. prepared a 6-membered supermacrocycle (rosette) by guanine and cytosine base derivatives, and they constructed helical autocatalytic rosette nanotubes (RNTs) through hierarchical self-assembly of these rosettes [28]. In response to water or methanol stimuli, right-handed and left-handed helical structures of RNTs were induced, respectively (from top or side view). Moreover, except solvent stimuli, the helical chirality inversion could be also achieved spontaneously or through thermal stimulus. Similarly, by a pair of enantiomeric solvents (*R*/*S*-limonene), helical chirality inversion of supramolecular nanotubes was achieved [29]. Mechanism studies revealed that the stereoselective interaction between the chiral monomer and *R*/*S*-limonene was the main driving force to modulate the helical senses of the supramolecular nanotubes.

**Figure 2:**
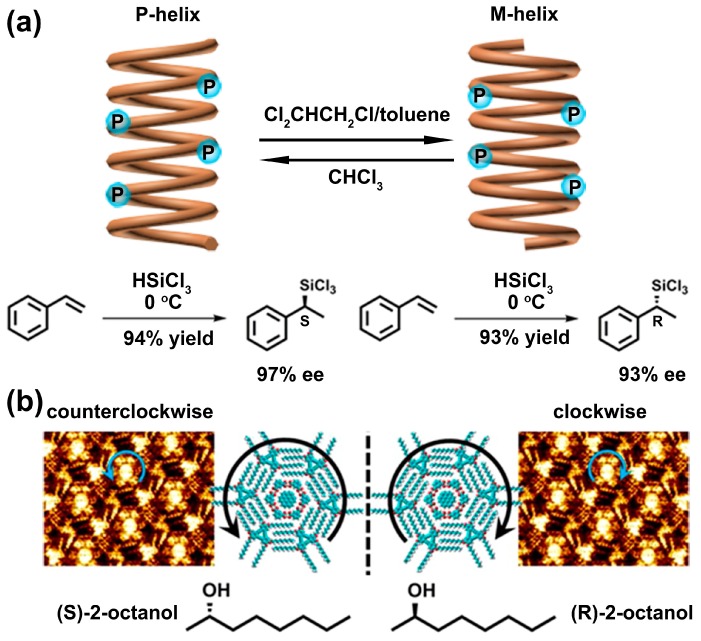
(**a**) Asymmetric hydrosilylation of styrenes using a PQXphos variant as ligand. Switching of enantioinduction in this reaction can be achieved by tuning the helix of the ligand via solvent stimuli; (**b**) DBA-OC10 can self-assemble into well-organized counterclockwise or clockwise nanopatterns at the (S)-2-octanol/highly oriented pyrolytic graphite (HOPG, left) and (*R*)-2-octanol/HOPG interface (right), respectively, observed by high resolution-scanning tunneling microscope (HR-STM). Reproduced from [37] with copyright permission.

In recent years, due to the easy separation from reaction mixtures as well as reusable possibility, polyquinoxaline-based phosphine (PQXphos) provides a highly effective scaffold for chiral catalysis. Via chiral modification of the side chains, PQXphos can form helical structures. Moreover, through organic solvent stimuli, helical senses of these structures can be inverted reversibly, hence creating a novel reaction environment with controllable helical chirality. This makes PQXphos a good candidate to construct a catalytical system capable of generating both enantiomeric products with high enantioselectivity. Suginome et al. published a series of studies on the construction of PQXphos materials as a macromolecular scaffold for asymmetric catalysis [21,30,31]. In 2010, the authors synthesized a kind of poly(quinoxaline-2,3-diyl)s bearing chiral (R)-2-butoxy side chains [32]. Interestingly, the authors found that helical sense of this polymer was solvent-dependent. In detail, the polymer exhibited a pure right-handed or left-handed screw sense in chloroform and 1,1,2-trichloroethane, respectively. Analogously, a variant of PQXphos with high-molecular-weight was also prepared by the authors [17]. Moreover, the character of solvent-induced helical chirality was used as a polymeric ligand in catalytic asymmetric hydrosilylation of styrenes. As shown in Figure 2a, the PQXphos variant adopts P-helical form or M-helical form in dichloromethane/toluene mixture and chloroform, respectively. Reversible helical sense inversion between the P-helical form and the M-helical form of the polymeric scaffold was further applied to switch the enantio-induction in hydrosilylation of styrenes, resulting in highly efficient enantio-discrimination (97% ee (*S*)-products and 93% ee (*R*)-products). Compared with small molecular catalysts, this novel polymer-based catalyst provided a relative new and simple method to selectively obtain production of either enantiomer with high enantioselectivity. Interestingly, events of solvent-induced chirality switching could be observed by the naked eye. In 2014, the authors fabricated dry solid polymer films by a PQXphos variant bearing (*S*)-2-methylbutyl, n-butyl and 8-chlorooctyl side chains [33]. After annealing in CHCl_3_ vapor, the solid films displayed reflection of right-handed circular polarized light (CPL). By contrast, chiral reversion of reflected CPL was observed after annealing in 1,2-dichloroethane vapor.

Helical chirality inversion of poly(phenylacetylene) through solvent stimuli has been also reported by Riguera et al. [34]. In 2010, they prepared a poly(phenylacetylene) bearing phenylglycine methyl ester groups. They found that the helical sense of this system exhibited solvent-responsiveness. CD spectra indicated that the polymer preferred to form left-handed helix in less polar solvents, and it tended to develop a right-handed helix in more polar solvents (i.e., DMSO). Subsequently, in 2013, they synthesized a similar poly(phenylacetylene) system, and found that the polarity of the solvent significantly impacted on the helical senses and the elasticity (stretching/compression) of the polymeric systems [35].

Notably, through solvent-stimuli, chirality switching was even realized on a solid surface, which was directly observed by means of atomic force microscope (AFM) and scanning tunneling microscope (STM). The chirality inversion at the interface shows profound significance due to numerous vital biological and chemical processes in vivo starting at the bio-interface, and mirrored conformational changes of a solid surface may markedly influence these processes. Yashima et al. prepared poly(phenylacetylene)s bearing l- or d-alanine residues side chain (i.e., poly-1d and poly-1l, respectively) [36]. Upon exposure to organic solvent vapors (e.g., tetrahydrofuran, chloroform), the polymers self-assembled into two-dimensional hierarchical structures on highly oriented pyrolytic graphite (HOPG). By varying solvent polarity, helical chirality inversion of the two-dimensional structure on HOPG could be achieved. For example, poly-1d generated a left-handed helix in polar solvents (e.g., chloroform), while it formed a right-handed helix in non-polar solvents (e.g., benzene), which were directly detected by high resolution AFM. In recent years, increasing attention has been focused on the induction of chirality in achiral monolayers instead of monolayers constructing by achiral molecules. Recently, Feyter et al. reported an achiral alkoxyalted dehydrobenzo annulene (DBA) derivative, in which the chirality was induced and switched via chiral solvents (e.g., (*S*/*R*)-2-octanol) [37]. In detail, the chirality of the generated honeycomb network was determined by the relative array of the four interdigitated alkyl chains per DBA pair (“+” or “−” type interdigitation). Combining six − type or + type interdigitation patterns would lead to virtual “clockwise” (CW) or “counterclockwise” (CCW) chirality respectively. Via high resolution STM (HR-STM), the authors found that CCW and CW honeycomb motifs of the compound on a HOPG surface were obtained upon self-assembly from (*S*)-2-octanol and (*R*)-2-octanol respectively, as displayed in Figure 2b. Besides, the solvent-induced chirality inversion was further verified by using a different pair of enantiomeric solvents (2-decanol). Compared with 2-octanol, the solvent impact on the chirality was more distinct in 2-decanol, especially in the presence of DBAs with longer alkoxy chains.

### 2.2. Photo-Directed Helical Chirality Inversion

In addition to solvent, photo-irradiation is another efficient factor to induce helical chirality inversion. Owing to the high temporal and spatial precision, light serves as a good stimulus source. The most simple and straightforward method to prepare photo-responsive systems is by introducing photo-switches (e.g., azobenzene, diarylethenes, dithienylethene, spiropyranes, etc.) into the systems. In response to light irradiation stimuli, large conformational transition of these photo-switches can be induced because of *trans*-*cis* isomerization, photocyclization, dimerization or photopolymerization processes. Hence, an incoming photo stimulus can lead to macroscopic property changes of the superstructures and even materials, which will offer significant advantages in the development of numerous molecular motors, molecular machines, and photo-responsive liquid crystals [38].

In recent years, much attention has been focused on light-induced chiral inversion, which may promote applications in the development of molecular devices, such as molecular motors, switches, gears, shuttles, and others. Feringa and coworkers designed a series of novel photo-responsive molecular devices, which were capable of converting light energy into directional rotary or linear movement [39]. In 2005, the authors constructed a novel light-driven motor, which consists of a rotor connected via a rotational axle to a stator part. The motor can be immobilized on a gold surface by the two terminated thiol groups of the stator part. In response to light or thermal stimuli, the system underwent four-state unidirectional rotation on the gold surface, accompanying inversion between M helicity and P helicity, which were demonstrated by circular dichroism (CD) and nuclear magnetic resonance (NMR) investigation.

**Figure 3:**
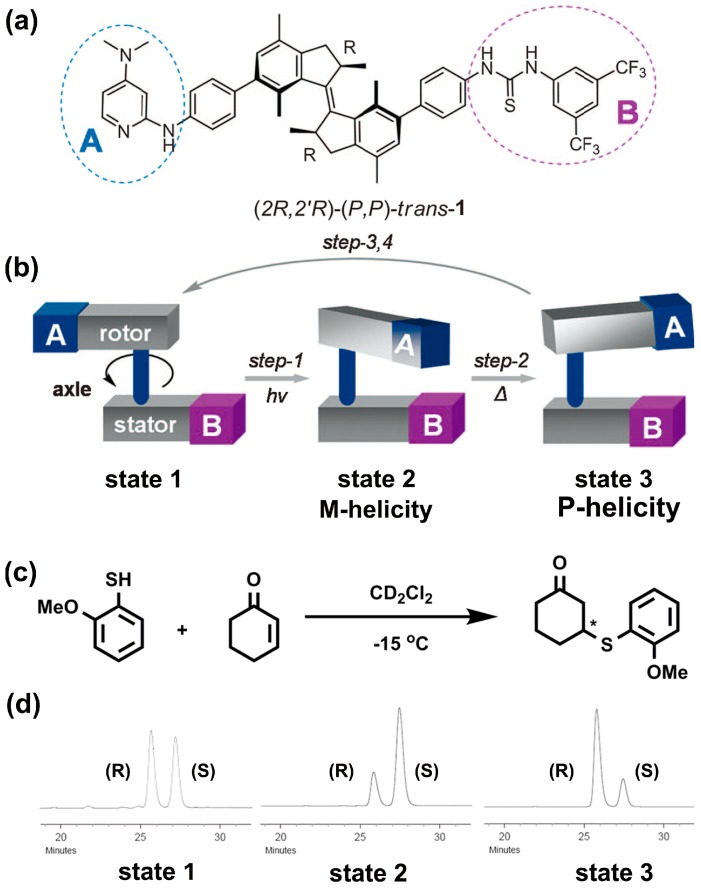
(**a**) Chemical structure of light-driven molecular motor; (**b**) Schematic illustration of one full rotary cycle by photo and thermally stimuli; (**c**) The catalytic reaction scheme and conditions; (**d**) HPLC traces of the reaction product utilizing catalyst in state 1 (*S*/*R* = 49/51), state 2 (*S*/*R* = 75/25) and state 3 (*S*/*R* = 23/77), respectively. Reproduced from [40] with copyright permission.

Chiral catalysts have been found to have broad applications to obtain single enantiomers with high enantiomeric excess (ee) value. However, it is still a big challenge to switch the chiral preference of a catalytic system in situ. Notably, in 2011, the same group solved this challenge by a light-responsive molecular motor with versatile catalytic functions, which can dynamically regulate the enantio-induction of target product in situ (see Figure 3) [40]. The position and helical orientation of the catalytic groups in this catalytic system were modulated by photo and thermal stimuli, thus producing three stages of this catalytic system with distinct chiral structures (i.e., state 1, state 2-M-helicity and state 3-P helicity). Interestingly, they found that the configuration of the system exhibited a significant impact on the catalyst performance in absolute stereo-control as well as catalytic activity. Either racemic, or preferentially the R or the S enantiomer of the target product could be obtained by tuning the system in state 1, state 2, and state 3 via light and thermal stimuli. Although the ee value of the product was not satisfactory, the light-induced chirality inversion has been successfully applied to switch the chiral preference of a catalytic system in situ.

Light-driven chiral switching of helical supramolecules or polymers has been also reported. In 2007, Pijper et al. prepared a helical poly-(n)-hexylisocyanate bearing a photo-responsive benzamide-functionalized motor positioned at the terminus of the polymeric chain [41]. In the absence of light irradiation, the polymer did not show any chiral preference for M or P helical sense. After the treatment with UV light irradiation, the terminal photo-responsive moiety underwent a *trans*-*cis* isomerization, which then led to a preferred M helix of the polymer backbone. Furthermore, after a thermal stimulus, the M helical structure changed into a preferred P helix because of thermal inversion of the terminal motor. In 2013, Govorov prepared a DNA origami bundle with helical structure, which was attached to a BSA-biotin-neutravidin-coated-surface [42]. Also, on the DNA scaffold, gold nanoparticles self-assembled into a left-handed helix. Interestingly, the assembled helical sense of the gold nanoparticles could be controlled by a polarized light stimulus, monitored through the analysis of CD spectra. Similarly, by a treatment of visible circularly polarized light (CPL), helical chirality of a polymer, consisting of achiral triphenylamine and diacetylene moieties, was switched selectively and reversibly [43]. Meanwhile, the induced helical sense of the polymer could be arrested after the treatment with ultraviolet light irradiation. Hence, in this polymeric system, induction, control as well as locking of supramolecular chirality could be achieved simultaneously. Similar studies have also been reported by Chen et al. [44] and Kim et al. [45].

For materials science, light-induced helicity inversion has also drawn profound attention. Specifically, helicity switching of LC media shows great significance in mimicking biological systems and fabricating fascinated three-dimensional materials and devices with controllable chirality [15]. A series of these LC materials was prepared by introducing photo-responsive chromophore guests (i.e., azobenzene, diarylethene) into achiral LC media hosts by Li et al. [15]. In 2010, the author prepared two cyclic azobenzenophane derivatives with axial chirality and introduced these photo-responsive units into three structurally different, achiral LC hosts [46]. By alternating UV light and visible light treatment, the photo-responsive units underwent *trans*-*cis* isomerization reversibly, which then triggered the helical handedness switching in the induced helical superstructures of LC hosts. Meanwhile, this photo-directed helix inversion event was observed by the naked eye since selective reflection colors of the LC materials changed from blue to near-infrared (NIR). Similarly, in 2012, the authors reported a pair of enantiomeric photo-responsive azobenzene switches, exhibiting a large conjugated system [47]. Notably, the *trans*-*cis* isomerization of the system was accomplished by visible light irradiation (440 and 500 nm) instead of UV light irradiation. By doping the photo-responsive switch in achiral LC media, a helical superstructure of the LC phase could be induced. For this azobenzene-based system, through visible light treatment, inversion of helical sense could be achieved, accompanied by distinct color reflections (i.e., red, green, and blue) of LC media. Furthermore, in 2015, the authors prepared a chiral diarylethene switch, which could induce the handedness inversion of LC helical superstructure via luminescence stimuli (λ: 808 and 980 nm) [22]. A core-multi-shell upconversion nanoparticle (NIR nanotransducer), capable of transducing dual wavelength NIR, was also synthesized. By the introduction of the photo-responsive switch (i.e., diarylethene) and the nanotransducer into the LC host, the handedness of the helical superstructures can be switched reversibly upon NIR light irradiation. Very recently, the authors advanced their research by using a novel cholesteric liquid crystals (CLC) system [48]. Through UV or Vis light stimulus, they realized helical chirality inversion of this CLC system. Moreover, three-dimensional manipulation of the helical axis was also achieved. Similar studies were also reported by Akagi et al. [49].

**Figure 4:**
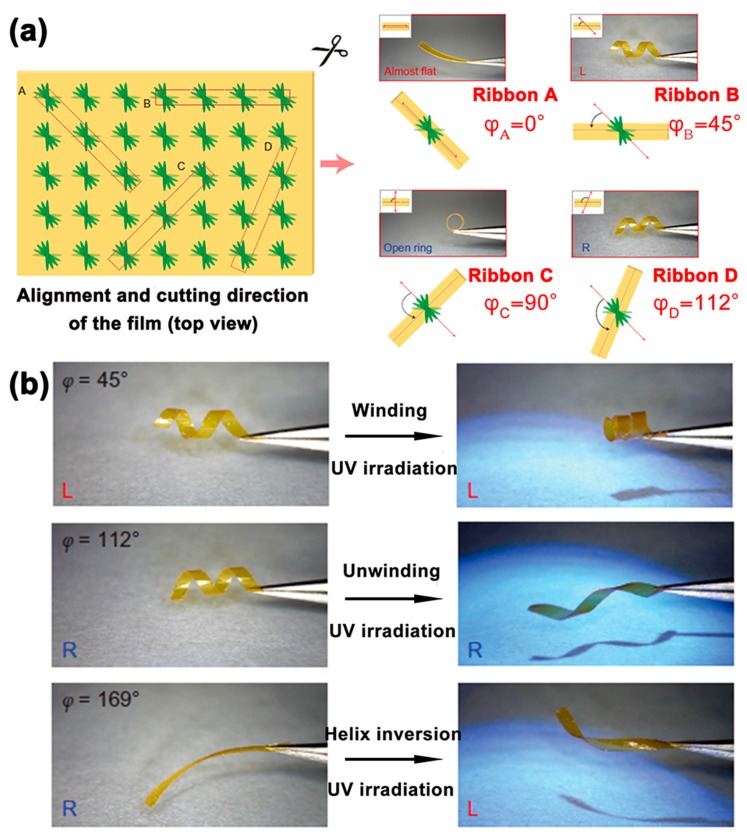
(**a**) Ribbons show distinct macroscopic shapes depending on the direction they are cut, ribbon A is almost flat, ribbon C exhibits an open-ring shape, ribbon B and D displayed left-handed or right-handed shape, respectively. (**b**) The mechanical motions (i.e., winding, unwinding, and helix inversion) can be observed by the naked eye via different ribbons triggered by UV light irradiation. Reproduced from [50] with copyright permission.

The ability to transform a light-induced helical inversion event into a macroscopic motional response of a material will offer a significant advantage in the development of artificial molecular machines, such as soft robotics, micromechanical systems, and artificial muscles. Meanwhile, conversion of light input into macroscopic movement of materials will allow us to detect the helical inversion directly. In 2014, Katsonis et al. designed a photo-responsive spring-like material, consisting of azobenzene-based switches and chiral dopants (i.e., *S*-811, *R*-811) embedded in liquid-crystalline polymer springs [50]. By introducing a small amount of *S*-811 or *R*-811 dopant, a left-handed or a right-handed twist in the liquid-crystalline mixture could be made, respectively. Taking the mixture containing *S*-811 dopant as an example, the mixture was then added into a glass cell to facilitate a twist geometry, and the orientation of the LC director changed smoothly from the bottom surface to the top surface. Interestingly, ribbons with different macroscopic shapes could be obtained by cutting the dried films in a different direction (the angular offset φ), as displayed in Figure 4a. Moreover, in response to UV light irradiation, different shapes of ribbons experienced distinct mechanical motions, such as winding, unwinding and helix inversion, which could be observed by the naked eye. Hence, winding and unwinding motions could be achieved simultaneously in a spring system containing mixed helical structures.

Interestingly, Omatsu et al. reported a novel method to switch the chirality of metal nanostructures (i.e., chiral metal nanoneedles) [51]. Instead of using photo-responsive switches to induce chiral inversion, they innovatively utilized an optical vortex laser to directly prepared chiral nanoneedles. Through just modulating the sign of the helical sense of the optical vortex, metal nanostructures with different chirality could be selectively obtained.

### 2.3. Ion-Directed Helical Chirality Inversion

Living systems have numerous evolved sophisticated proton-mediated conformational switches, which play key roles in a series of vital chemical and biological processes. Inspired by nature, chemists and materials scientists have designed a variety of ion-responsive systems, of which the helical chirality can be tuned by cation or anion stimuli [52].

**Figure 5:**
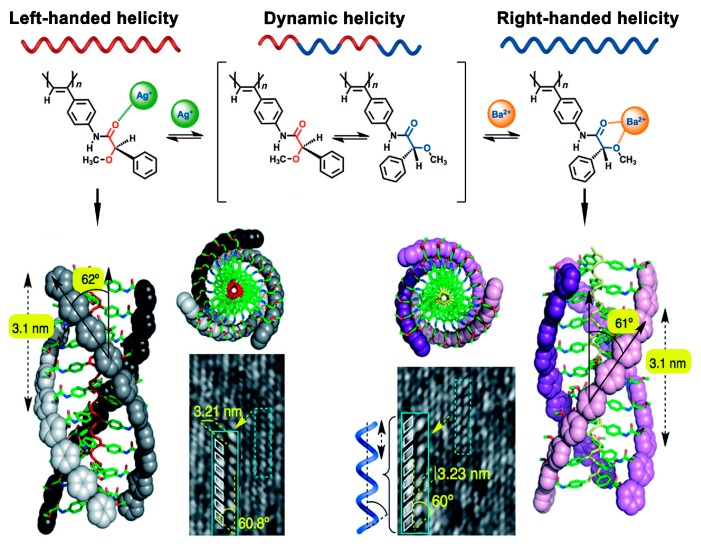
Scheme illustration of selective regulation of the helix sense by control of the conformation of the pendants upon Ag^+^ or Ba^2+^, respectively (upper part); AFM images and theoretical models (form the top and side views) of left-handed helix of polymer/Ag^+^, or right-handed helix of polymer/Ba^2+^ (lower part). Reproduced from [23] with copyright permission.

One of the most widely used methods for ion-directed conformational change is protonation, and a series of studies on cation-mediated chirality inversion have been reported. Riguera et al. performed a series of investigations on the construction of helical supramolecular assemblies from poly(phenylacetylene)s (PAAs). In 2010, the authors synthesized a PAA bearing phenylglycine methyl ester groups, induction and inversion of helical sense of this polymer could be achieved by choosing appropriate metal cation species as stimuli [34]. By analyzing the CD spectra of the polymer after the treatment of five perchlorates of mono- or divalent metal cations (i.e., Li^+^, Na^+^, Ag^+^, Mg^2+^ and Ba^2+^), the authors found that all of these cations could trigger the helical chirality inversion of this polymer, and Ba^2+^ generated the strongest effect. Subsequently, the authors prepared a PAA derivative bearing a chelating unit (α-methoxyphenylacetic acid). The authors investigated the influence of the addition of perchlorates of mono and divalent metal cations on the formation of helical senses of the polymer [23]. Without ion stimuli, the polymer was in a highly dynamic state, which contained both left-handed and right-handed assemblies without predominant helicity. Interestingly, the chiral side chains interacted with mono or divalent cations differently, which then induced left- or right-handed predominant helicity, respectively, as displayed in Figure 5. This phenomenon could be directly detected by AFM images of the polymer combined with Ba^2+^ or Ag^+^ on a highly oriented pyrolytic graphite surface. Hence, this polymer can be used as a novel sensor for the detection of mono and divalent cations. In 2014, the same group also realized the switching of the helical senses of a copolymer system by ion stimuli (i.e., Li^+^ and Ba^2+^) [53]. Interestingly, in 2015, the authors achieved selectively either left-handed helix or right-handed helix using only one ion (i.e., Na^+^) [54]. Recently, they advanced their research by preparing a highly dynamic poly(arylacetylene) system [55]. They found that the polymer could form helically controlled nanostructures (e.g., nanospheres, nanotubes, toroids) by interaction with ions (i.e., Li^+^, Na^+^, Ag^+^). With Ag^+^, the polymer preferred to form nanospheres with M helicity and tunable sizes. In contrast, with Li^+^ and Na^+^, the polymer preferred to yield nanotubes, gels or toroids with M helicity as well as encapsulating properties. Analogously, a similar work using metal ions to switch macroscopic chirality of chiral nanostructures has also been reported by the same group [56].

Nabeshima et al. also reported a cation-responsive host-guest system [57]. A helical tetranuclear complex containing two benzocrown units was prepared and used as molecular leverage to induce and switch helical sense. By introducing alkanediammonium guests, the helical sense of the host can be modulated. The authors found that the guest with a short alkyl chain promoted the formation of *P*-helical isomer, while, the guest with a long alkyl chain preferentially caused a helical inversion to M-helical isomer. Similar works using cation to trigger helix inversion were reported by Jung et al. [58] and Haberhauer et al. [59].

Studies of anion-dependent helical inversion have been also reported. Miyake et al. synthesized a peptide-metal complex, consisting of a chiral metal center bearing two achiral peptide arms [60]. The helix inversion of the peptide-metal complex was achieved by the introduction of an achiral NO^3−^ anion. Through electrostatic interaction, NO^3−^ anion stimulus first triggered helical chirality inversion of the chiral metal center, which then caused helical inversion of the two achiral peptide arms. In 2011, Suk et al. prepared a novel chiral indolocarbazole dimer capable of realizing helicity inversion upon anion binding [61]. The circular dichroism spectra indicated that helicity inversion could be completed by the addition and removal of anions (i.e., SO_4_^2−^).

### 2.4. pH-Directed Helical Chirality Inversion

The pH value is another convenient stimulus resource for modulating helical chirality.

In early studies, Tang et al. prepared a polyacetylene bearing l-valine side chains [62]. Driven by hydrogen bonds among polymeric chains, the l-valine pendants promoted the formation of a helical structure. CD spectra indicated that the helicity of the polymer chain could be modulated reversibly by the addition of KOH, or the neutralization of the solution with HCl. Transformation between helix and coil can also be realized by adjustment of pH value. In 2010, Aida et al. prepared an oligo(4-aminopiperidine-4-carboxylic acid) (Api8, Api: an achiral, non-natural amino acid bearing basic side chain), the helix to coil transition of this oligopeptide could be reversibly induced by tuning the pH value of the corresponding medium [63]. In acidic media, protons interacted with the basic side chains, promoting the formation of an H-bonding network, consequently, leading to a helical conformation of the oligopeptide. In sharp contrast to this, in a pH range of 7–10, the induced helical sense turned into a random coil conformation due to the break of the original H-bonding network. Similar work was also reported by Tanaka et al. [64]. Vibrational circular dichroism (VCD) spectroscopy is a quite reliable method for studying chirality of molecules. By adjustment of pH value, the helical sense of a supramolecular system in insulin fibrils reversed, which was directly observed by VCD and AFM [65].

More interestingly, inspired by the helical handedness inversion between right-handed B-DNA and left-handed Z-DNA in vivo, artificial DNA-templated assemblies exhibiting tunable helical handedness have also been prepared. Schenning et al. designed a host-guest system, consisting of DNA host template and achiral naphthalene derivative guest [24]. The achiral guest was equipped with a diaminopurine (owning a quite large π surface), that easily coupled with the DNA template. At pH = 3, this host-guest system tended to form a left-handed helicity; while, at pH = 7, this system was inclined to form a right-handed helicity. Mechanism study indicated that the chirality switching was attributed to the protonation of the achiral guest. At low pH values, the protonated guest molecule stabilized the host-guest complex with a left-handed structure; at pH = 7, the host-guest tended to be more positive, resulting in a right-handed conformation.

### 2.5. Thermo-Directed Helical Chirality Inversion

As a quite ubiquitous stimulus in nature, temperature also provides a convenient method to modulate the helix of supramolecular and polymeric systems.

Control of spatial conformation (e.g., α-helix) of peptide-based systems shows great significance because it enables a better understanding of the pathological pathways of many neurodegenerative diseases, such as Parkinson’s and Alzheimer’s diseases. Based on a theory of the non-covalent chiral domino effect (NCDE), Inai et al. realized the helical sense switching of a nonapeptide by thermal stimulus [66]. The nonapeptide was mainly constructed of eight achiral, non-natural amino acids (i.e., Z-α,β-dehydrophenylalanine and α-aminoisobutyric acid) with a chiral Ala head. Through NCDE, the molecular chirality of the Ala head was transferred and amplified into the helical chirality of the nonapeptide. Besides, by lowering or increasing the temperature of the nanopeptide solution, the helical sense changed between left-handed and right-handed reversibly. In 2012, Meijer et al. developed a chiral supramolecular through the self-assembly of achiral oligo(p-phenylenevinylene) ureidotriazine (AOPV3) [67]. AOPV3 just formed racemic helical stacks without complexation of chiral additive (i.e., l- or d-tartaric acid (l- or d-TA)). When AOPV3 combined with l- or d-TA, a preferred helical structure could be induced because of the strong interactions between the chiral additive and AOPV3, which was indicated by the mirror CD spectra of AOPV3 in the presence of l-TA or d-TA. In addition, the induced helical chirality maintained after removing the chiral additive. Besides, the supramolecular system exhibited thermo-responsiveness. At room temperature, the helical structures were quite stable for a long time, however, the preferred helicity turned into racemic helicity when the temperature of the solution was raised to approximately 60 °C. In addition, symmetry breaking occurred through cooling the solution, thus leading to the recovery of the helical senses of the supramolecular system. Similarly, Ajayaghosh et al. reported a thermally assisted inversion of an azobenzene-based supramolecular handedness, which was directly observed by SEM and AFM images [68]. Interestingly, Yamaguchi et al. designed a pseudoenantiomeric aminomethylenehelicene oligomer, which formed three states with distinct conformations (i.e., right-handed helix, left-handed helix, and random coil) during reversible thermodynamic processes [69]. In general, traditional three-state molecular switching systems usually involve multiple stimulus methods. By comparison, the system in this study achieved structural switching reversibly among three different states by just employing a single thermal stimulus.

**Figure 6:**
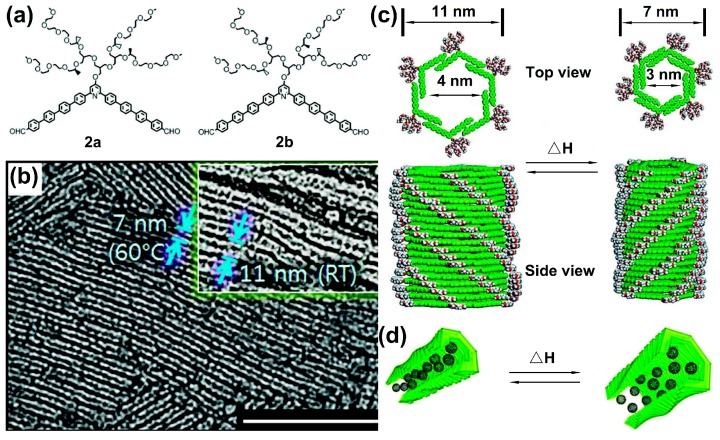
(**a**) Molecular structures of enantiomeric bent-shaped amphiphiles 2a (left) and 2b (right); (**b**) TEM images of 2a tubules (0.01 wt % aqueous solution) prepared at 60 °C or room temperature (inset), scale bar: 100 nm; (**c**) Schematic illustration of pulsating rotation of the nanotubules accompanied by a chiral inversion, triggered by using thermal stimuli; (**d**) Schematic illustration of thermo-modulated packing variations of encapsulated C_60_ molecules. Reproduced from [25] with copyright permission.

Yashima and coworkers reported a series of studies on chirality inversion of helical polymers (e.g., polyacetylene) through thermal stimulus. In 2005, they designed a *cis*-transoidal poly(phenylacetylene) bearing a bulky phenyl phosphonate group, which exhibited a dual memory of enantiomeric helices [70]. By addition of chiral additive, the polymer assembled into the preferred helical structure. Taking (*R*)-1-(1-naphthyl)ethylamine ((*R*)-2) as an example, the polymer preferred a left-handed helix upon combining with (*R*)-2 at 25 °C, whereas, it turned into a right-handed helix when the temperature was increased to 65 °C, indicated by the analysis of the CD spectra. In addition, the induced helical sense was “frozen” even after the removal of (*R*)-2. Later, the authors prepared a series of co-poly(phenylacetylene)s bearing optically active cyclodextrin (CyD) residues, including α-CyD, β-CyD, γ-CyD, and permethylated β-CyD (denoted as poly-1α, poly-2β, poly-3γ, and poly-2β-Me, respectively) [71]. Helical chirality inversions of these polymeric systems, together with obvious color changes of the corresponding solutions, were also achieved by other external stimuli, such as temperature, solvent or guest molecules (i.e., 1-phenylethylamine). More interestingly, in 2012, the authors designed novel supramolecular nanotubules, which experienced a reversible helical inversion along with contraction-expansion motions through thermal stimulus, as displayed in Figure 6 [25]. A pair of enantiomeric bent-shaped rod amphiphiles were prepared (Figure 6a, denoted 2a and 2b); 2a (0.02 wt % aqueous solution) formed elongated tubules with a uniform external diameter of approximately 11 nm and internal diameter of approximately 4 nm at room temperature, as observed in the TEM image (Figure 6b). In response to a thermal stimulus, adjacent aromatic segments underwent a remarkable slide, which then caused pulsating motions of the helical tubules accompanied by a helix inversion (Figure 6c). Moreover, due to the shrinkage and swelling of the tubules (Figure 6d), the thermo-induced dynamic motion of the tubules then led to the release of different amounts of encapsulated C_60_ guests.

## 3. Bio-Application of Helicity-Controllable Polymer

Taking advantages of sophisticated structures and functions in the helical supramolecular structure, various artificial chiral materials and devices have been developed and have found a wide range of applications in drug delivery, imaging, biosensors, asymmetric catalysis, molecular switches, responsive liquid crystal materials, and other related areas. Helicity-controllable polymeric systems classified by the category of polymer are listed in Table 1.

Studies have proved that a helical structural motif in a helical polymer is a critical factor for achieving efficient drug delivery. Kataoka et al. reported a core-shell polymeric micelle loaded with platinum drugs. This micelle consists of cisplatin-conjugated poly(l-(or d-)glutamate) (P(l (or d)Glu)-CDDP as the core and poly(ethylene glycol) (PEG) as the shell [72]. Circular dichroism (CD) studies showed that right-handed and left-handed helical nanostructures in the core of cisplatin-loaded micelles (CDDP/m) were induced by P((l)Glu) and P((d)Glu) blocks, respectively. In contrast, the core of CDDP/m exhibited an achiral nanostructure by optically inactive P((d, l)Glu) building blocks. Through chloride ion stimulus, the release of cisplatin was triggered, which then led to the disassembly of the CDDP/m because of the increased electrostatic repulsion as well as the decreased hydrophobicity. The antitumor efficacy of racemic d,l-CDDP/m decreased markedly because the micelle structure gradually disintegrated in the bloodstream. In sharp contrast, l-CDDP/m maintained its micelle structure via helical bundles of the l-CDDP/m core during circulation, thus keeping the micelle structure for a long time and achieving more therapeutic efficiency against pancreatic tumor. Zhong et al. prepared an efficient delivery vesicle of doxorubicin hydrochloride (DOX·HCl) by a pH-responsive triblock copolymer-poly(ethylene glycol)-*b*-poly(l-leucine)-*b*-poly(l-glutamic acid) (PEG-PLeu-PGA) [16]. When the pH value decreased from 7.4 to 5.0, the random coil structure of PGA blocks changed into an α-helix structure. Such a structural change led to the collapse of pepsomers, hence significantly enhancing the release efficiency of DOX·HCl. A quite similar pH-responsive vesicle was also reported by Bellomo et al. [73].

Helical polymers also show prospect for potential application in imaging. Wu et al. prepared a pair of poly (γ-benzyl l-glutamate)-poly (4-cyano-benzoic acid 2-isopropyl-5-methyl-cyclohexyl ester) rod-rod diblock copolymers bearing l- or d-menthyl pendants (denoted as PLGA-PPI (l) and PLGA-PPI (d)) [74]. Because of the chiral induction of the pendants, the two copolymers self-assembled into nanofibrils with opposite helical senses. Interestingly, PLGA-PPI (l) exhibited a much higher encapsulation of chiral d-amino acid modified rhodamine chromophores (RhB (D)) over PLGA-PPI (d), which then rendered PLGA-PPI (l) a better performance in live HepG2 cell imaging.

**Figure 7:**
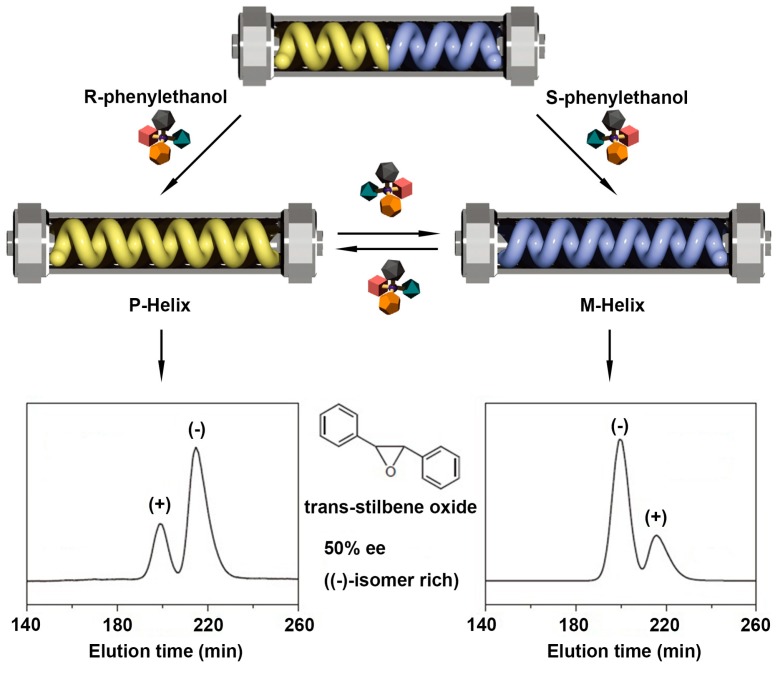
Schematic illustration of switchable enantioseparation (trans-stilbene oxide) by poly-1 stationary phase. Through *R*/*S*-phenylethanol stimulus, the original racemic helicity can turn into P-helix or M-helix, generating a great impact on the elution order of trans-stilbene oxide enantiomers. Reproduced from [75] with copyright permission.

Besides, novel materials for chiral recognition and separation can also be prepared based on a helicity-controllable polymeric system. Maeda et al. designed and prepared a stationary phase by a polyacetylene with 2,2’-bisphenol derived side chains (poly-1) [75]. Distinct interactions between *R*/*S*-phenylethanol and the polymer side chains induced the original racemic helical stationary phase into P-helix or M-helix, as displayed in Figure 7. In addition, after removing the chiral alcohols, these induced helical conformations were maintained due to a memory effect. Based on this solvent-directed helical chiral inversion, switchable enantioseparation of trans-stilbene oxide was achieved by poly-1 stationary phase. In detail, the elution time of (+)-*trans*-stilbene oxide was shorter than that of (−)-*trans*-stilbene oxide when the stationary phase exhibited P-helical structure. By comparison, the elution order reversed when the M-helical structure of the stationary phase was used.

Another important challenge, as well as a new and promising direction of materials with tunable helicity is how to tune the helical chirality of biomolecules (i.e., DNA and proteins), or use biomolecules as building blocks to construct novel chiral materials, such as cholesteric LC materials. Tracking this challenge will not only enable us to better understand the elegant manipulating mechanism of bio-molecular superstructures capable of yielding switchable helical chirality in vivo, but also guide us to design and prepare chiral materials to mimic the unique structures and functions of biomolecules. Viruses are natural supramolecular structures consisting of coat protein assemblies, which have been widely used in biomedicine, biotechnology, and energy [76]. Notably, in recent years, researchers have used biological viruses as building blocks to construct cholesteric LCs, exhibiting macroscopic helical chirality. Dogic et al. used two filamentous viruses (i.e., *fd* wide-type (wd) and *fd* Y21M) to build cholesteric LCs [77]. The two rod-like viruses only differed in one amino acid residue in the major coat protein pVIII. Interestingly, LC materials, constructed by these two viruses, exhibited opposite handedness and distinct helical pitch. The *fd* Y21M LCs formed a right-handed helix with a helical pitch five times larger than that of *fd* wt LCs. In contrast, *fd* wt LCs exhibited a left-handed helix. Moreover, a LC system with tunable macroscopic helical chirality was obtained by adjusting the ratio of the two viruses in their mixture. Lee et al. advanced this investigation from a different perspective [78]. The authors used s single type of bacterial virus (M13 phage) to prepared varied chiral LC M13 phage films by means of dip coating methods. By precisely controlling pulling speed, phage concentration, surface chemistry of M13, and the solid support, LC M13 phage films with distinct supramolecular structures, such as cholesteric helical ribbons, nematic orthogonal twists, or smectic helicoidal nanofilaments, could be prepared. These M13 films displayed uniquely different optical and photonic properties, which promoted their potential applications as displays or reflectors. Furthermore, these films were then utilized as versatile templates to guide cells to grow in multiple directions in a hierarchically organized manner. This work achieved the transformation from helical biomolecules to hierarchically structured materials. This inspires us to design and prepare tunable hierarchically structured materials by employing a system with tunable helical chirality as a template structure.

## 4. Conclusions and Outlook

In this mini-review, we outlined the recent progress of a variety of molecular and polymeric systems which experience reversible helix inversion upon external stimuli, including solvent-, light-, ion-, pH- and thermo-stimuli. In these systems, helical senses can be switched reversibly on receiving external stimuli in an intelligent mode. These mirrored-helix inversions can be also converted and amplified into changes in macroscopic properties of the corresponding materials, which then bring a diverse range of applications, such as drug delivery, biosensor, chiral catalysis, smart optical and electronic systems, photo-responsive LC materials, intelligent molecular devices, micromechanical systems, and microfluidic devices. In addition, given the great significance of helicity inversion in many physiological processes, this mini-review is designed to enlighten us on the design and preparation of biomaterials and biomimetic materials with controllable helicity.

However, this research field is still in its infancy, numerous challenges remain to be solved. First, from the perspective of responding to variety and accuracy, multi-responsive systems are quite attractive since they could respond to more than one stimulus, which will endow sophisticated responsiveness to the corresponding materials, and promote the development of smart and biocompatible materials. However, to date only a fairly limited number of multi-stimuli responsive systems have been produced. In addition, it is also interesting to develop creative systems that can respond to different stimuli, such as biochemical signals (e.g., specific biomolecules or biochemical reaction) [79,80]. Second, although many fantastic mirrored-helicity inversion investigations have been reported, can we go further? In the aspect of the modulation of molecular conformation, can we realize reversible switching between the α-helix and the β-sheet of peptides and proteins. The achievement of that will not only enable a comprehensive understanding on protein amyloidosis but also provide novel insights to treat neurodegenerative diseases. Third, to develop the practical nanodevices and nanomachines, the transition of molecular helix inversion is usually too weak or too slow to maintain continuous operation of a nanodevice. Hence, more versatile systems, capable of translating and amplifying weak molecular conformational changes into distinct variations of materials macroscopic properties, should be fabricated.

## Figures and Tables

**Table 1:** Helicity-controllable polymeric systems classified by the category of polymer. Stimulation, regulating mechanism as well as typical references are included.

Polymers	Stimulation	Regulating mechanism	Ref
Poly(phenylacetylene)	Solvent	The effect of solvent polarity	[34,35,36]
	Ion	Cation–π Interactions	[56]
	pH	Electrical repulsion	[62]
	Temperature	Noncovalent helix induction	[70,71]
Poly(peptide)	Ion	Chirality transfer	[60]
	-	*Cis-trans* isomerization	[11]
	Temperature	Chiral domino effect	[66]
Poly(quinoxaline)	Solvent	The effect of solvent polarity	[21,30,31,32,33]
Poly(diphenylacetylene)	Solvent	Chirality transfer	[27]
Poly(hexylisocyanate)	Light	Photoisomerization	[41]
